# The relationship between attachment, primary emotions and positive/negative spirituality: a path analysis

**DOI:** 10.3389/fpubh.2024.1375850

**Published:** 2024-06-26

**Authors:** Anton Freund, Jürgen Fuchshuber, Giorgia Silani, Human-Friedrich Unterrainer

**Affiliations:** ^1^Institute of Psychology, University of Vienna, Vienna, Austria; ^2^Department of Psychoanalysis and Psychotherapy, Medical University Vienna, Vienna, Austria; ^3^Comprehensive Center for Clinical Neurosciences and Mental Health, Medical University Vienna, Vienna, Austria; ^4^Institute of Psychology, University of Graz, Graz, Austria; ^5^CIAR: Center for Integrative Addiction Research, Grüner Kreis Society, Vienna, Austria; ^6^Department of Psychiatry and Psychotherapeutic Medicine, Medical University of Graz, Graz, Austria; ^7^Department of Religious Studies, University of Vienna, Vienna, Austria; ^8^Faculty of Psychotherapy Science, Sigmund Freud University, Vienna, Austria

**Keywords:** attachment, religious/spiritual wellbeing, spiritual struggles, path analysis, primary emotions

## Abstract

**Objective:**

The present study investigates what may influence individuals to experience their religiosity/spirituality as either subjectively positive [religious or spiritual (r/s) wellbeing] or as negative (r/s struggles). Drawing on existing literature attachment insecurity and the seven primary emotions as outlined by Jaak Panksepp in Affective Neuroscience are identified as likely influences.

**Methods:**

The final sample consisted of 340 participants (age: *M* = 36, SD = 14.2; 68.5% = female), among which 65% self-identified as religious/spiritual. A path analysis was conducted to test a proposed mediation model in which the expected effects of primary emotions (B-ANPS) on r/s wellbeing (MI-RSWB) and r/s struggles (RSSS) were mediated through attachment insecurity (ECR-RD8).

**Results:**

The data indicated that attachment insecurity fully mediated the relationships between the primary emotions SADNESS and LUST with r/s struggles. Furthermore, the primary emotions FEAR and ANGER displayed small direct effects on both r/s struggles and r/s wellbeing. Overall, the model, which demonstrated excellent model fit, was able to explain 30% of the variance of r/s struggles, 24% of attachment insecurity and 5% of r/s wellbeing.

**Conclusions:**

The findings suggest that primary emotions such as SADNESS and LUST substantially explain r/s struggles and that these relationships seem to be mediated through attachment. Moreover, r/s struggles seem to be qualitatively distinct from r/s wellbeing. Finally, a moderate link between LUST and attachment suggests that sexuality plays a significant role in (adult) attachment processes.

## Introduction

In the recent past attention and interest in the discipline of Psychology of Religion and Spirituality (1) has increased considerably and reflects a new awareness of the ubiquity–84% of the world's population consider themselves religious (2)—and influence religion and spirituality (r/s) have on human behavior and health (3).

Spirituality refers to a search for the sacred, namely, aspects in life connected to the divine or transcendent. Religion, on the other hand, constitutes a broader context of institutions, teachings, and communities that aim to facilitate their members spirituality (2, 3). Psychology of Religion and Spirituality (Division 36, APA) focuses on studying the influences of r/s on human behavior across cultures and has established itself as a thorough scientific discipline, often surpassing other fields of psychology in methodological soundness (4).

As research on the effects of r/s on various human health aspects has grown in the past two decades, it became clear that r/s may have largely positive and salutogenic effects, yet it may in some cases have negative or even pathogenic effects (5, 6). Thus, r/s can be a protective factor and additional resource that helps individuals cope with challenges (6) leading to positive health outcomes, such as lower suicide rates (7, 8), increased longevity (9) and lower rates of depression (10). However, r/s may also correlate with poorer health outcomes in certain instances, including increased stress (10, 11), depression (12, 13) and other psychopathologies (14).

### Religious/spiritual wellbeing and religious/spiritual struggles

In correspondence to this, r/s may express itself internally as a positive or negative subjective quality, which has been conceptualized psychologically as r/s wellbeing or r/s struggles (15, 16). Firstly, r/s wellbeing refers to a sense of wellbeing in relation to a “non-physical dimension of awareness” often referred to as God or the “sacred” (16). Yet, it is often seen as a multifaceted variable with transcendent (e.g., a positive relation to God, sense of divine presence, hope in afterlife) and existential/immanent elements (e.g., hope in a good future, sense of purpose, forgiveness) (17). Conversely, r/s struggles represent distress, internal conflicts, or negative emotions related to beliefs, practices, or experiences connected to the sacred (18). These struggles may manifest in various ways such as divine struggles, e.g. fear or anger toward God; demonic struggles, as fear of demonic attacks; interpersonal struggles, as with one's r/s community; and intrapersonal struggles, which includes moral dilemmas, doubts, and the search for meaning.

Research exploring the individual reasons behind the development of either r/s struggles or r/s wellbeing has mostly focused on elements such as r/s involvement (10), r/s practices, such as meditation (18), or religious coping styles (19). This study wants to contribute to the growing field of Psychology of Religion and Spirituality by further investigating the internal psychological conditions in which r/s tends to express itself positively (as r/s wellbeing) or negatively (as r/s struggles).

### Attachment insecurity and the divine

The attachment system (20) might offer a potential trajectory in understanding the individual development of r/s wellbeing or struggles (21).

It evolved in mammals to regulate the proximity between caretaker and the vulnerable infant, thus increasing the survival chances of the offspring.

A child that feels unsafe may engage in attachment behaviors such as crying or raising its arms to prompt the caregiver to establish physical proximity (e.g. by picking up the child) and sooth it. Moreover, to regulate their own felt security children create mental representations of the parents, enabling them to endure the absence of the caretaker without distress and fearing abandonment. These mental representations become embedded in Internal Working Models (IWMs) which constitute models and expectations of their parents' availability and responsiveness to their needs. They reflect early attachment experiences and are the foundation for the attachment style or attachment security of the child.

According to Bowlby, the attachment system is active over an individual's entire lifespan (“from the cradle to the grave”) [(20), p. 207] and many relations can have an attachment function, as e.g. romantic relationships (22) and friendships (23), even online friendships (1).

Moreover, once a child develops symbolic thinking capacities it may actively imagine invisible figures with an attachment component (e.g. imagined friends) (22, 23). In a similar manner, the attachment system may project itself onto an invisible or symbolic figure such as a God or other transcendent entities (24). Finally, an individual's attachment security will, due to its continuity across relations (20), influence many relationships that serve an attachment purpose including a relation with a transcendent entity such as God (25, 26).

The parallels between real-world attachment relations and one with a transcendent entity are multifold and may illustrate the applicability of the attachment system on r/s (27).

To begin with, many individuals view a “personal relationship” with God as the essence of religion (28) and in Christianity God is explicitly referred to as *Father* and believers as *God's Children* (21, 27). Freud already viewed God as a projection of an infantile wish for an all-powerful father figure (29). Moreover, proximity regulation is a key function of the attachment system for which God might be ideal in its conceptualisation as omnipresent, thus, always available. Yet, by going to a *house of God* as a church or a temple or through prayer individuals claim to subjectively experience God's presence and proximity (30). In prayer one may even raise the arms like a child that wants to be picked up. Finally, the usually practiced “relationship with God” meets the five attachment criteria outlined by Ainsworth (32) as God is often experienced as a “safe haven” and a “secure base” from which to explore the world with confidence (31). For a more detailed analysis see Granqvist and Kirkpatrick (33).

Attachment security tends to affect wellbeing and satisfaction across relations (33, 34) and may affect the relation to the transcendent or God in a similar way (21). Due to the continuity of the attachment pattern and IWMs (35) the securely attached individual is likely to experience God as supporting and loving as well (20). The literature refers this as the correspondence hypothesis (36).

While many religions describe God in ways that fits the description of an ideal caretaker (being loving, comforting and guiding), God may also embody the negative and frightening aspects an attachment figure can have.

He could, for example, punish one for one's sins, be indifferent to one's fate or even abandon the believer (damnation). This is like the actual parent/caretaker, who may be punishing, unavailable or even abandoning. These parallel and divergent aspects could suggest, why r/s may be a source of strength for some and a source of distress for others. Depending on one's attachment security one may expect God to be loving and supportive, leading to r/s wellbeing, or unavailable, cold and harsh, resulting in r/s struggles (37).

### Primary emotions and attachment

The system of primary emotions (PE), as outlined by Panksepp (39), impacts the attachment system in distinct ways (39, 40) and may, therefore, indirectly influence whether r/s is expressed as r/s wellbeing or r/s struggles. Like all mammals own an expression of the attachment system, they also share similar PE systems (38). These systems consist of positive PEs such as CARE, PLAY, SEEK and LUST as well as the negative PEs like ANGER, FEAR and SADNESS (see Table 1). The corresponding affective brain-circuits are assumed to reside in older and lower parts of the brain, can be switched on/off via deep electrical brain stimulation (ESB) (42) and continue to function even after decortication (43). Moreover, there is some evidence suggesting that PEs may be “anchoring” and influencing personality in a bottom-up fashion (44). Finally, the various PEs might influence the attachment system in a similar bottom-up fashion (41).

**Table 1:** The seven primary emotional systems as outlined by Panksepp.

**Primary emotions**	**Evolutionary function (attachment focus)**
PLAY	Developing social skills via pretend-like practices; encourages social engagement and bonding
CARE	Securing survival of offspring; providing comfort and support; promotes secure attachment in offspring
SEEK	Driving curiosity and exploration of environment; promoting interaction with caregivers
FEAR	Activation of fight/flight system ensuring survival; motivates to seek safety from caregivers when threatened or distressed
ANGER	(Re-)claiming resources, distress signal
SADNESS	Separation distress; encourages reconnecting or repairing bond to attachment figure
LUST	Sexuality; promoting attachment bonds between romantic partners

Davis and Panksepp's (41) seven primary emotional systems with focus on attachment.

**Figure 1:**
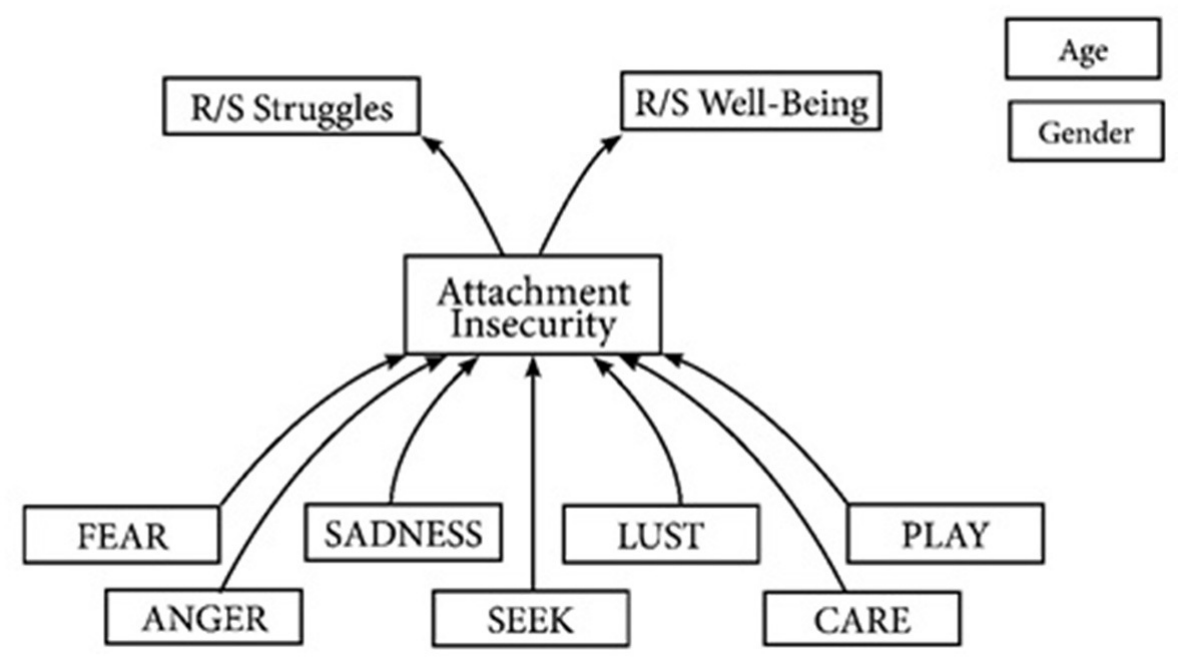
Path analysis model of associations between primary emotions, attachment, R/S wellbeing and R/S struggles controlled for age and gender.

The PEs with potentially the greatest influence on the attachment system may be SADNESS (45). SADNESS, occasionally labeled as the “separation distress-system” (41), refers to the profound feelings of grief and panic associated with the loss or absence of an attachment figure and thereby motivate reestablishment of contact or proximity. Yet, other PEs may influence the attachment system as well. LUST, for example, may play a significant role in the building of trust and intimacy in adult attachment relations (e.g., romantic partnerships) and FEAR may amplify the perceived need of protection from attachment figures. For a comprehensive list see Table 1. Moreover, previous research suggests that PEs influence r/s via personality (46). However, no study to date has investigated the influence of PEs on r/s wellbeing and r/s struggles via attachment functions within a single model. To address that gap, this subsequent study will evaluate the effects of the different PEs on r/s wellbeing and r/s struggles via attachment within a mediation model (see Figure 1).

### Hypotheses and mediation model

The present study investigates a model (see Figure 1) in which PEs serve as independent variables influencing r/s wellbeing and r/s struggles via attachment insecurity. It is hypothesized that both negative and positive PEs, particularly FEAR, SADNESS and LUST, exert independent effects on attachment insecurity. Furthermore, attachment insecurity is expected to show a negative association with r/s-wellbeing and a positive association with r/s struggles. Finally, we expect attachment insecurity to mediate indirect effects of the PEs on r/s wellbeing and r/s struggles.

## Method

### Participants and procedure

The sample exceeded the required sample size (*N* = 310), as determined with a G^*^Power analysis (34, 35), which aimed at a power of 0.8 (for conservative estimates small effect sizes were assumed). The initial sample included 348 participants (67.8% female) ranging in age from 18 to 78 years (*M* = 36.3, *SD* = 14.27). Moreover, participants self-identified with various religious (48%), spiritual (14%) or secular belief systems (29%). This was measured with a single item (e.g. “Do you feel affiliated with a religion? If yes, to which? If not, do you consider yourself spiritual, atheistic, agnostic or none of the above?”). Further demographics assessed included highest completed level of education, vocational field, relationship status, number of children and fluency in German. Finally, the survey, which included 98 items, took on average 14 min to complete.

Furthermore, recruitment was conducted using university mailing lists, online forums, and personal networks between December 2022 and May 2023. As incentive participants were invited to partake in a drawing of one of 10 book-store vouchers worth €10 each. Moreover, data collection was carried out via the online platform SoSci Survey. Finally, through partial listwise deletion, eight participants that missed out on at least one entire variable were removed. Yet, participants that missed only single items were retained in the analysis.

### Religious/spiritual wellbeing (MI-RSWB 18)

To assess r/s wellbeing the *MI-RSWB 18* (47) was administered, a validated and abbreviated version of the original *MI-RSWB 48* (16). The *MI-RSWB 18* is an 18-item questionnaire measuring r/s wellbeing across six dimensions each consisting of three items. Considering recent empirical findings, a comprehensive factor (RSWB) was determined via the subscales General Religiosity (GR) and Connectedness (CO) (48). This factor concentrates on r/s wellbeing in relation to the transcendent (example item: *In certain moments I feel very close to God*).

Concurrently, the remaining subdimensions serve as supplementary factors in the overarching model. Moreover, the items were rated on a 6-point Likert scale indicating the extent to which an item applied to them ranging from 1 (*does not apply at all*) to 6 (*applies to a great degree*). Lastly, in line with previous research (47) the general factor (RSWB) demonstrated very good reliability with a Cronbach's alpha of 0.88 (see Table 3).

### Religious and spiritual struggles

R/s struggles were assessed using the German version of the *Religious and Spiritual Struggles Scale* (*RSSS*) (1) originally developed by Exline et al. (15). The *RSSS* consists of 26 items and measures r/s struggles over six domains. These subscales seek to capture internal conflicts and tensions with regards to specific dimensions of religious or spiritual life. They include (1) divine struggles (*felt as though God had let me down*), (2) demonic struggles (*felt attacked by the devil or by evil spirits*), (3) interpersonal struggles (*felt rejected or misunderstood by religious/spiritual people*) and intrapersonal struggles, such as (4) moral struggles (*worried that my actions were morally or spiritually wrong*), (5) doubts around one's beliefs (*felt confused about my religious/spiritual beliefs)* or (6) struggles around ultimate meaning (*felt as though my life had no deeper meaning*). Furthermore, participants rated the degree to which an item applied to them using a 5-point Likert scale ranging from 1 (*does not apply at all*) to 5 (*applies to a great degree*). The total score was calculated as the average score of all 26 items. Finally, with an Cronbach's alpha of 0.90 the RSSS total score exhibited excellent reliability (see Table 3).

### Attachment (ECR-RD8)

To assess the adult attachment security of the participant, the self-report questionnaire *Experiences in Close Relations—Revised (ECR-RD8)* was implemented (49). The response format is a 6-point Likert scale indicating the extent to which participants agree with a given statement from 1 (*do not agree*) to 6 (*totally agree)*. Moreover, the *ECR-RD8* follows a dimensional conceptualisation in which attachment style is a function of the subscales *avoidance* (e.g. *I find it easy to depend on romantic partners*) and *anxiety* (e.g., *I often worry that my partner will not want to stay with me*) (50) measured with four items each. However, to analyse the data with respect to a single dimension of attachment (*attachment insecurity*), the scores of the two subscales are summed up indicating the extent of general attachment insecurity (higher scores indicating more insecure attachment). Finally, with a Cronbach's alpha of *0.8*1 the total score of the of the *ECR-RD8* displayed good internal consistency (see Table 3).

### Primary emotions (BANPS-GL)

The BANPS-GL (51) is a self-report questionnaire that measures the seven emotional systems known as Primary Emotions (PE) developed within the framework of Affective Neuroscience (39). These include the negative emotional systems FEAR (*I sometimes cannot stop worrying about my problems*), ANGER (*When I am frustrated, I usually get angry*), and SADNESS (*I often feel lonely*) and the positive emotional systems CARE (*I often feel the urge to nurture those closest to me*), SEEK (*I enjoy finding new solutions to problems*), PLAY (*I am a person who is easily amused and laughs a lot*) and LUST (*For me, it is easy to indulge myself in erotic experiences*). The 38-item questionnaire uses a 5-point Likert scale ranging from 1 (*totally not agree*) to 5 (*totally agree*). Moreover, the BANPS-GL is a translated and validated version of the BANPS (52). In addition, it includes a recently developed LUST-scale (53) making it the first questionnaire to measures all PE scales. Finally, the PEs SADNESS, LUST, FEAR, PLAY, and ANGER demonstrated acceptable to very good reliability with α-coefficients between 0.74 and 0.87. Yet, the CARE and SEEK subscales had poor to questionable Cronbach's alpha of 0.55 and 0.63 (see Table 3).

### Statistical analysis and analysis plan

For data organization, descriptive statistics, bivariate correlations as well as assumption testing SPSS 29.0 was utilized. Firstly, the data was cleaned by removing participants that did not complete the questionnaire or miss out on at least one entire variable (partial listwise deletion). Followingly, means, internal consistencies and intercorrelations of the variables were inspected. Moreover, a test of multivariate normality indicated no violation (critical ratio = 0.489), thus, *p*-values for direct effects were not bootstrapped.

To test the proposed mediation model a path analysis was conducted with AMOS 28. The initial model included (1) paths from all seven primary emotions to attachment, (2) paths from attachment to both r/s wellbeing and r/s struggles, and finally, (3) a direct path from each primary emotion to r/s wellbeing and r/s struggles (see Figure 1). Additionally, to account for the potential confounding effects of gender and age, these were also included in the model. Existing literature indicates that age and gender significantly influence one's r/s (54, 55). Finally, after the model was fitted a pruning strategy was applied in which all nonsignificant paths between variables and between disturbance terms were removed.

Goodness of fit was assessed using the maximum likelihood estimation method. As benchmarks for an acceptable fit, a set of fit indexes were considered following the general guidelines set out by Kline (56). These included (a) the chi-square to degrees of freedom ratio (CMIN/DF) < 3; (b) the comparative fit index (CFI) >0.90; (c) the Tucker-Lewis index (TLI) >0.90 and, lastly, (d) the square root error of approximation (RMSEA) < 0.08 including an upper bound of < 1 (in the 90% confidence interval).

Subsequently, due to the relatively small sample size, to test for indirect effects, a bootstrap was performed with a bias-corrected confidence interval of 95% and 2,000 bootstrap samples (bias corrected percentile method) (57). Furthermore, modification indices were checked, and variables and disturbance terms allowed to covary if theory justified it. The alpha level was set to *p* < 0.01.

## Results

### Sample characteristics

After removal of eight participants due to missing data using partial listwise deletion, the sample contained 340 participants. Of this final sample *n* = 233 (68.5%) were female and their ages ranged from 18 to 78 years (*M* = 36.3, *SD* = 14.27). Almost half of the participants (*n* = 169, 49.8%) reported to be religiously affiliated, a third (*n* = 102, 29.7%) considered themselves secular (atheistic or agnostic) and *n* = 48 (14.1%) self-identified as spiritual. Christians were the largest group *n* = 142 (41.8%) and represented 84% of all religiously affiliated participants. Education-wise, most participants reported to have at least a bachelor's degree (*n* = 232, 68.2%). Moreover, *n* = 110 (32.4%) were students and social workers constituted the largest professional group (*n* = 95, 27.9%). Finally, while most participants were in a relationship or married (*n* = 215, 63.8%), a majority was also without children (*n* = 241, 70.9%). For detailed sample characteristics, see Table 2.

**Table 2:** Background characteristics of participants.

**Sample characteristics**	**(*N* = 340)**
**Age, years**	
*M* (SD)	36.3 (14.27)
Range	18–78
**Gender**, ***n*** **(%)**	
Male	100 (29.4)
Female	233 (68.5)
Other	7 (2.1)
**Religious affiliation**, ***n*** **(%)**	
Christianity	142 (41.8)
Islam	14 (4.1)
Buddhism	6 (1.8)
Hinduism	1 (0.3)
Other	6 (1.8)
Spiritual	48 (14.1)
Atheist	61 (17.9)
Agnostic	41 (12.1)
No option applies	22 (6.5)
**German skills**, ***n*** **(%)**	
Fluent	334 (98.2)
Basic skills	6 (1.7)
**Educational level**, ***n*** **(%)**	
Secondary education	10 (2.9)
High school diploma	85 (25)
Vocational training	18 (5.3)
Bachelor	130 (38.2)
Master	89 (26)
PHD	13 (3.8)
**Vocation**, ***n*** **(%)**	
Craftmanship	6 (1.8)
Social profession	95 (27.9)
Medical profession	16 (4.8)
Business profession	60 (17.6)
Academic profession	39 (11.5)
Student	110 (32.4)
Unemployed	10 (2.9)
**Relationship status**, ***n*** **(%)**	
In partnership/married	215 (63.2)
Single/divorced	125 (36.8)
**Number of children**, ***n*** **(%)**	
No children	241 (70.9)
One child	27 (7.9)
Two or more children	71 (20.9)

### Descriptive statistics

For all variables, the number of items, internal consistencies (α- and ω-coefficients), means, standard deviations, ranges and inter-correlations are displayed in Table 3.

**Table 3:** Number of items, internal consistencies, means, SDs and ranges of variables.

**Scale**	***N* items**	**Cronbach's α**	**McDonald's ω**	***M***	**SD**	**Range**
RSSS total	26	0.90	0.89	1.94	0.67	2.77
RSWB	6	0.88	0.89	60.67	10.09	61
ECR	8	0.81	0.79	20.32	8.74	42.49
SADNESS	6	0.87	0.87	16.67	5.50	24
LUST	5	0.81	0.81	18.97	4.09	20
FEAR	5	0.81	0.80	15.76	4.46	20
ANGER	6	0.74	0.74	14.22	4.45	21
PLAY	6	0.79	0.79	22.73	4.33	20
CARE	4	0.55	0.55	16.09	2.58	13
SEEK	6	0.63	0.61	23.10	3.69	17

RSSS total, Religious and Spiritual Struggles Scale total score; RSWB, General Factor of the Multidimensional Inventory of Religious and Spiritual wellbeing; ECR, Experiences in Close Relationships combined score.

### Inter-correlations

The single largest intercorrelation was found between the primary emotions SADNESS and FEAR (*r* = 0.62). Furthermore, many correlations were moderately large (between *r* = 0.30 and *r* = 0.40; Cohen, 1992). This included but was not limited to the intercorrelations between LUST and the ECR (*r* = 0.40), between FEAR and the RSWB (r = 0.39), and between SADNESS and RSSS (*r* = 0.38). Finally, the primary emotions PLAY, CARE, and SEEK had overall very few significant correlations. An overview of all intercorrelation can be found in Table 4.

**Table 4:** Pearson's intercorrelations between RSSS total, RSWB, ECR, and BANPS-GL (primary emotions).

**Scale**	**RSSS total**	**RSWB**	**ECR_8**	**SADNESS**	**LUST**	**FEAR**	**ANGER**	**PLAY**	**CARE**
RSSS total	–								
RSWB	−0.12^*^	–							
ECR	0.37^**^	−0.29^**^	–						
SADNESS	0.38^**^	−0.23^**^	0.37^*^	–					
LUST	−0.24^**^	0.18^**^	−0.40^**^	−0.24^**^	–				
FEAR	0.30^**^	−0.39^**^	0.28^**^	0.62^**^	−0.16^**^	–			
ANGER	0.28^**^	−0.21^**^	0.18^**^	0.2^**^	−0.09	0.22^**^	–		
PLAY	−0.16^**^	0.05	−0.24^**^	−0.21^**^	0.32^**^	−0.10	−0.03	–	
CARE	−0.09	0.11	−0.14^*^	0.00	0.28^**^	0.10	−0.08	0.36^**^	–
SEEK	−0.04	0.06	−0.06	−0.11	0.21^**^	0.08	−0.03	0.20^**^	0.17^**^

RSSS total, Religious and Spiritual Struggles Scale total score; RSWB, General factor of the Multidimensional Inventory of Religious and Spiritual wellbeing; ECR, Experiences in Close Relations overall insecurity score; BANPS-GL, Brief Affective Neuroscience Personality Scales including a LUST scale.

^*^p < 0.05.

^**^p < 0.01.

### Model pruning

**Figure 2:**
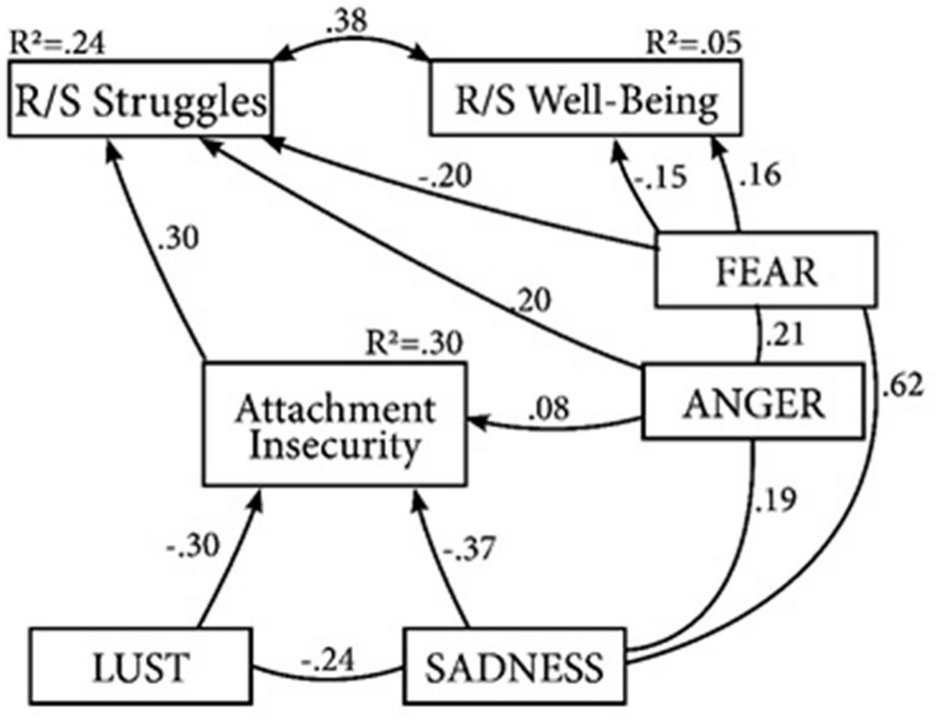
Final model controlled for age and gender. PEs affecting R/S struggles and R/S wellbeing directly and via attachment insecurity. Lines display significant associations with *p* < 0.01.

The original model (Figure 1), which was corrected for the effects of age and gender, contained both direct and indirect effects of primary emotions on attachment, r/s wellbeing, and r/s struggles. The initial model was saturated and exceeded the acceptable threshold for the chi-square to degrees of freedom ratio (CMIN/DF = 15.82). Thus, to improve model fit and achieve model parsimony, a pruning strategy was put to task in which nonsignificant paths were removed: firstly, (1) nonsignificant paths from SEEK, PLAY, CARE to r/s wellbeing, r/s struggles and attachment; (2) paths from LUST and SADNESS to r/s wellbeing and r/s struggles and lastly (3) the path from FEAR to attachment were removed. Additionally, disturbance terms of r/s wellbeing and r/s struggles were allowed to covary due to their conceptual relatedness. Thus, the resulting model (Figure 2) contains only significant paths, and the primary emotions PLAY, SEEK and CARE were removed due to the lack of significant associations. For model fit indices, see Table 5.

**Table 5:** Model fit indices: initial and final model.

**Models**	**df**	**χ^2^**	***p***	**CFI**	**TLI**	**RMSEA (90% CI)**
Initial model (Figure 1)	222	348.10	>0.01	0.46	0.10	0.21 (0.20; 0.23)
Final model (Figure 2)	316	28.62	>0.05	0.98	0.95	0.05 (0.02; 0.08)

Df, degrees of freedom; χ^2^, chi-square; CFI, Comparative Fit Index; TLI, Tucker-Lewis Index; RMSEA, Root Mean Square Error of Approximation; 90% CI, 90% Confidence Interval.

### Direct effects

Moderate positive direct effects were seen between SADNESS (β = 0.37; *p* < 0.001) as well as LUST (β = −0.30; *p* < 0.001) and attachment insecurity. Furthermore, small directs effects of ANGER on RSWB (β = 0.15; *p* = 0.007) and on RSSS (β = 0.20; *p* < 0.001) were observed. Similarly, FEAR displayed small negative direct effects of RSWB (β = −0.20; *p* < 0.001) as well as a small positive direct effect on RSSS (β = 0.16; *p* < 0.01). Furthermore, attachment insecurity showed a moderate direct effect on RSSS (β = 0.30; *p* < 0.001). Finally, small direct effects were observed from the control variables Age (β = −0.11; *p* < 0.01) and Gender (β = 0.13; *p* < 0.01) on RSSS indicating that men and older people report higher r/s struggle scores.

### Indirect effects

A bootstrap analysis revealed two statistically significant indirect effects of primary emotions mediated by attachment insecurity on r/s struggles. The observed effects include a small positive indirect effect of SADNESS on r/s struggles (β = 0.11; *p* = 0.001) and a small negative indirect effect of LUST on r/s struggles (β = −0.09; *p* = 0.001). Finally, the analysis revealed no indirect effects between primary emotions and r/s wellbeing mediated by attachment insecurity.

### Overall model

The trimmed model (Figure 2) displayed excellent model fit: CMIN/DF = 1.79, CFI = 0.98, TLI = 0.95, RMSEA = 0.05 (90% CI: 0.02; 0.08) (56). In summary, this model was able to explain 30% of the variance of attachment insecurity, 24% of the variance of r/s struggles and 5% of the variance of r/s wellbeing. The effects of the primary emotions FEAR and LUST on r/s struggles were fully mediated by attachment, partially supporting the hypothesis. However, effects of primary emotions on r/s wellbeing were not mediated by attachment. The hypothesis that primary emotions effect r/s wellbeing via attachment is thus not supported by the data.

## Discussion

### Summary of findings

The main objective of this study was to assess what factors influence an individuals' experience of religion/spirituality as either positive (r/s wellbeing) or as negative (r/s struggles). Constituting the central finding of this study, the PEs SADNESS and LUST indirectly affected r/s struggles via attachment insecurity. However, no PEs affected r/s wellbeing indirectly via attachment insecurity. Instead, FEAR and ANGER affected r/s wellbeing and r/s struggles directly. Finally, the model explained a substantial degree of attachment insecurity and r/s struggles, yet only a small proportion of r/s wellbeing. Thus, the findings support our suggested mediation model with regards to the indirect influence of SADNESS and LUST on r/s struggles.

### Theoretical considerations

As hypothesized and in accordance with prior research (58, 59), individuals with more attachment insecurity experienced higher levels of r/s struggles. This provides further evidence for the correspondence hypothesis (37) suggesting that the continuity of the attachment pattern extends to the divine. Thus, how one experiences real-world attachment relations such as partnerships, friendships or family relations tends to translate to the experience of a symbolic attachment figure. Negative assumptions about the attachment figure and about oneself inform internal working mechanisms that increase the likelihood of inner tensions and conflicts with regards to the transcendent. Thus, fears of a punitive God or of God's abandonment may be more likely if the individual feels deserving of punishment or prior attachment relations were emotionally unavailable or unresponsive. Finally, the association between attachment insecurity and r/s struggles supports the application of attachment theory on religious and spiritual matters and warrants more nuanced analyses of the relation between the scales' subdimensions.

However, in contrast to our hypothesis and unlike r/s struggles, attachment insecurity was not associated with less r/s wellbeing as measured with the *MIRSWB-18* (60). This could suggest distinct mechanisms affecting r/s wellbeing and r/s struggles. The small negative association between r/s wellbeing and r/s struggles (see Table 4) suggests that despite their conceptual relatedness they represent distinct underlying realities and do not mirror each other. Moreover, the model explained a very small variance of r/s wellbeing, indicating that other variables which were not incorporated into this model may be more instrumental in the development of r/s wellbeing. All in all, the distinct pathways, low negative correlation with r/s struggles, and low proportion of explained variance point to a qualitatively different development of r/s wellbeing in comparison to r/s struggles.

Notably, the effects of SADNESS on r/s struggles were fully mediated by attachment insecurity. Sadness and grief are intimately linked with the attachment system and often the result of actual or imagined loss (45, 61). SADNESS—generally experienced as painful—motivates the individual to reconnect and get close to the caretaker replacing feelings of loss or separation with a sense of connection and closeness. Likewise, SADNESS may motivate the religious or spiritual person to reconnect with God or a Higher Power, thus, relieving the painful emotion by establishing a sense of connection and protection to such an attachment surrogate (21, 62). Yet, individuals with more insecure attachment may have more difficulty finding a “haven of safety” or guidance in another person and likewise within one's religion or spirituality. Thus, SADNESS may remain high and contribute to a sense of r/s struggles.

Further, individuals with higher scores in LUST/sexuality reported less r/s struggles through more secure attachment. LUST, in contrast to SADNESS, is a positive PE and Panksepp and Biven (43) describe it as an emotional system linked to pleasure, sexual urges and gratifications as well as to social aspects such as bonding and sexual attachments. The strongest attachment relationships for adults are usually romantic partnerships and sexual intimacy plays a crucial role in that bonding process. Considering that our sample contains only adults it may not surprise to see the size of the association between LUST and attachment to be almost as large as that between SADNESS and attachment. In a way, SADNESS could be seen as a negative, painful, non-sexual and LUST as a positive, pleasure-related, sexual component of adult attachment.

Finally, higher scores in FEAR were connected to more r/s wellbeing and less r/s struggles. Contrastingly, higher scores in ANGER resulted in less r/s wellbeing and in more r/s struggles. This suggests that FEAR and ANGER affect r/s struggles directly. However, their effects were small and not mediated by attachment insecurity.

### Limitations

Due to the cross-sectional study design our findings do not warrant causal interpretations. I.e., the model fit had remained the same if the roles of the independent variables (PEs) and the mediator (attachment insecurity) were interchanged. However, the chosen model was based on theoretical considerations as the Triune Brain Theory by MacLean (63) which plays a significant role in the understanding of PEs (64). Yet, to be able to make causal inferences temporal precedence between PEs, attachment insecurity and r/s struggles needs to be established via longitudinal study designs.

Second, as a path analysis approach was chosen measurement error could not be accounted for. Therefore, the reported associations are most likely slightly underestimated, which could have been avoided using Structural Equation Modeling (SEM) (56, 65). This concerns especially the PEs CARE and SEEK which show low internal consistency (Table 4) and were removed from the final model.

Furthermore, the sample size was too small to conduct detailed multi-group analyses. It had been valuable to analyse whether the effects differed across various religious, spiritual, and secular groups. Moreover, it had enabled more detailed analyses regarding the subscales of the RSSS (r/s struggles) and the MI-RSWB (r/s wellbeing). Another important limitation involves the absence of control items which would have decreased the likelihood of response bias. Finally, the study was not preregistered in the Open Science Framework, which is slowly becoming a new standard in Psychology (66).

## Conclusion

This study was to the authors knowledge the first to propose a mediation model in which primary emotions affect r/s struggles and r/s wellbeing via the attachment system. The findings suggest that the individual's experience of r/s as struggles is in part shaped by PE systems and attachment insecurity. On the one hand, this information may help clinicians dealing with clients that suffer from r/s struggles by considering attachment experiences and emotional factors, especially regarding SADNESS and LUST, in the therapeutic process. On the other hand, a valuable research trajectory could be to investigate the relationships between r/s struggles, attachment and PEs using neuroimaging data (e.g., fMRI).

Interestingly, our data indicates that r/s wellbeing was not affected as hypothesized and seems to develop in a distinct way compared with r/s struggles. This may suggest that r/s wellbeing is qualitatively different from r/s struggles. Thus, r/s struggles may not be the opposite or the absence of r/s wellbeing just as health is not merely the absence of illness (67). Furthermore, our model explained only a very small variance of r/s wellbeing suggesting that other variables not included in our model may be more important in the development of r/s wellbeing. Future research may elucidate that, especially with bigger sample sizes that could enable detailed analyses of subscales.

Finally, an interesting discovery of this study was a substantial connection between LUST/sexuality and attachment. An attempt to explain this relation was offered in the possible role of sexuality in the attachment process particularly in adults (romantic relationships). However, research on LUST/sexuality and attachment or even r/s is sparse. This may be due to measurement concerns (41). Yet, a potentially lingering sense of taboo regarding the scientific study of LUST/sexuality may have discouraged thorough scientific inquiry. All in all, the findings of this study indicate that (1) LUST/sexuality and SADNESS are closely linked to the attachment system, and they (2) affect r/s struggles via attachment security. These findings open new trajectories in developing an understanding of how r/s struggles come about.

## Data availability statement

The raw data supporting the conclusions of this article will be made available by the authors, without undue reservation.

## Ethics statement

The studies involving humans were approved by Ethics committee of the University of Graz. The studies were conducted in accordance with the local legislation and institutional requirements. The participants provided their written informed consent to participate in this study.

## Author contributions

AF: Writing – original draft. JF: Writing – review & editing. GS: Writing – review & editing. H-FU: Writing – review & editing.
